# Dynamic Smoothing, Filtering and Differentiation of Signals Defining the Path of the UAV

**DOI:** 10.3390/s22239472

**Published:** 2022-12-04

**Authors:** Aleksey S. Antipov, Julia G. Kokunko, Svetlana A. Krasnova, Victor A. Utkin

**Affiliations:** V.A. Trapeznikov Institute of Control Sciences of Russian Academy of Sciences, 65 Profsoyuznaya Street, 117997 Moscow, Russia

**Keywords:** UAV path planning, path smoothing, signal filtering and differentiation, sigmoid function

## Abstract

On the example of a control system for an unmanned aerial vehicle, we consider the problems of filtering, smoothing and restoring derivatives of reference action signals. These signals determine the desired spatial path of the plant at the first approximation. As a rule, researchers have considered these problems separately and have used different methods to solve each of them. The paper aims to develop a unified approach that provides a comprehensive solution to mentioned problems. We propose a dynamic admissible path generator. It is constructed as a copy of the canonical control plant model with smooth and bounded sigmoid corrective actions. For the deterministic case, a synthesis procedure has been developed, which ensures that the output variables of the generator track a non-smooth reference signal. Moreover, it considers the constraints on the velocity and acceleration of the plant. As a result, the generator variables produce a naturally smoothed spatial curve and its derivatives, which are realizable reference actions for the plant. The construction of the generator does not require exact knowledge of the plant parameters. Its dynamic order is less than that of the standard differentiators. We confirm the effectiveness of the approach by the results of numerical simulation.

## 1. Introduction

In automatic control systems for various technical plants and mobile robots, it is often necessary to solve an auxiliary problem of processing sensor signals and external influences. For example, sensor signals often contain measurement noise, which requires their preliminary filtering. The reference actions determine the path of an unmanned moving plant and can enter the control system in real time from an autonomous source. They must be filtered, differentiated, and smoothed. The quality of functioning of the entire automatic control system depends on the methods used to solve these problems. Therefore, the development of efficient and real-time signal processing methods is an urgent task. Let us consider the key aspects of each of these problems and the existing methods for their solution.

In automatic control systems with feedback, various controllers are used: linear and non-linear, continuous and discontinuous. The selection of controller depends on the purpose of control and priori information about the plant and environment for its operation. Many controllers are quite sensitive to noise, which affects the quality of control. In order to solve the problem of filtering measurements, the Kalman filter is traditionally used. It also performs the function of a state observer if the set of sensors is not complete. Note that, when estimating, it is fundamentally impossible to separate the useful signal from the noise in the case when they operate in the same frequency band. Under certain conditions, the Kalman filter can be optimally tuned according to the criterion of minimum mean square estimation error [1]. The implementation of the Kalman filter requires exact knowledge of the parameters of the plant model, or their adequate identification, which is not always possible. In some cases, it is possible to restore unmeasurable derivatives of output signals without using a dynamic model of the control plant. One can use numerical differentiation based on the calculation of finite differences [2], or more complex computational algorithms [3]. However, these methods are not efficient for noisy measurements, as they lead to a delay in the system and increase the noise amplitude. Using them, estimation errors accumulate with an increase in the order of the restored derivative. The use of multidimensional low-pass filters also generates a delay [4], which can lead to the loss of stability of a closed-loop system. Thus, the differentiation of signals using dynamic models is preferable.

In this paper, we consider the problem associated with estimating not the state variables of the control plant, but the derivatives of the reference actions. These signals enter the control system from an autonomous source. There is no analytical description of reference actions; only their current values are known. This situation arises in control systems for unmanned mobile robots. For the synthesis of a high-precision tracking system and the formation of control (as a function of a time or in the form of feedback), it is necessary to restore the derivatives of the reference actions. The order of the required derivatives is equal to the relative degree of the system (the number of differentiations of the controlled variables that must track the reference actions before the appearance of control). In the deterministic case, with a small relative degree of the system, methods of numerical differentiation of signals can be used to restore the derivatives of the reference actions. As noted above, it is advisable to use dynamic differentiators to obtain high-order derivatives.

The principle of constructing and tuning a dynamic differentiator is similar to solving the problem of observing unmeasured state variables of a control plant. The difference is that the differentiator restores the state variables of the virtual canonical system with an undefined input. The output of this system is the reference signal. Its state variables are the derivatives of the reference signal to be restored, and the unknown input is the next derivative [5]. On the basis of this system, one construct state observers of various structures with various corrective actions. They ensure the convergence of the observer-differentiator variables to the output signal and its unmeasured derivatives. In the presence of noise, an undefined input is an obstacle to tuning the observer-differentiator for filtering purposes. It is interpreted as an external bounded disturbance, which requires the use of special methods to suppress it.

The standard solution is to use a linear observer with high gains [6,7,8]. It has a fairly simple tuning; the settling time of the estimation error is controlled by just one parameter that defines a hierarchy of high gains. In order to reduce the noise component, one introduces low-pass filters into the observation loop [9]. The main drawback of an observer with high gains is the peaking phenomenon. This effect is related to the overshoot of estimating signals that arise due to the unboundedness of linear corrective actions.

In [10], Levant has developed the second order sliding mode observer with discontinuous corrective actions bounded in absolute value. Due to this observer, one can obtain estimates of derivatives in finite time without large overshoot. Hybrid observers are known [11], in which corrective actions contain both linear and discontinuous functions. However, the use of discontinuous controls leads to the problem of chattering (when an unwanted high-frequency component is present in the received estimated signals). In order to eliminate chattering, in particular, higher-order sliding modes are used [12,13]. Here, the order of the sliding mode must be at least one greater than the order of the estimated derivative. An alternative method is to approximate the sign function by a linear function with saturation [11,14,15,16]. Such observer-differentiators combine the advantages of linear observers with high-gains and sliding mode observers, but are free from their drawbacks.

One can conclude that the problem of restoring the derivatives of the measured signals has been studied quite fully, including in the presence of noise. However, in this paper, the subject to be processed are the reference actions for technical control plants. These signals may require not only filtering but also smoothing. The reference actions track the output variables of mechanical and electromechanical plants. Therefore, they determine the allowable movement in the workspace and be implemented by the plant. Thus, the given path of a mobile robot on a plane or in space must be sufficiently smooth. It must have bound continuous curvature since the robot cannot instantly change its movement direction. The problem of admissible trajectory planning is a separate, rather time-consuming task. As a rule, it is solved off-line, using spline interpolation or complex geometric calculations [17,18,19,20,21,22,23,24,25]. If there is reason to believe that the given curves are smooth and realizable, then one can solve the problem of restoring their derivatives and filtering by the above methods (using dynamic differentiators with special tuning). In this paper, we assume that the given path is constructed at the first approximation and is not smooth. For example, it could be a polyline connecting waypoints. Therefore, an additional problem arises, to smooth the reference action signals in real time, considering the constraints on the velocity and acceleration of the control plant. Note that conventional dynamic differentiators do not have a smoothing effect. The reason is that the differentiator works as a state observer. It is tuned so that the differentiator variables reproduce the measured signal and its unmeasured derivatives as accurately as possible.

The hypothesis of this study is that it is necessary to use a tracking differentiator [26,27] for simultaneous smoothing and restoration of derivatives. It has the input-output canonical form. The input (corrective action) is a stabilizing function that depends on the tracking error (residual between the reference action and the output of the tracking differentiator).

Using this concept, we have developed a dynamic generator of realizable reference actions with S-shaped corrective actions. The generator allows us to complexly solve the problems of restoring derivatives, filtering and smoothing signals. Note that in papers devoted to tracking differentiators, the problem of smoothing the processed signal is not posed. As a rule, only the first two problems are considered.

We demonstrate all the constructions on an example of an unmanned aerial vehicle (UAV) control system in flight mode. A specific plant was selected to detail the developed approach. Without loss of generality, one can apply it to other automatic control plants.

Section 2 describes a model for the spatial motion of the UAV center of mass. We propose a typical control law that ensures that the center of mass follows a given spatial path. The problem of information support of the control law is formalized in terms of processing the vector signal of the reference action: (i) restoring its first and second derivatives, (ii) filtration, and (iii) smoothing in real time, considering the constraints on the velocity and acceleration of the control plant.

Section 3 presents the main results of this paper. Section 3.1 substantiates the structure and method for synthesizing a dynamic generator capable of providing a comprehensive solution to the posed problems. Section 3.2 presents a procedure for synthesizing a dynamic generator. This procedure ensures that the output variables of the generator track a given non-smooth, non-noisy signal with some accuracy. It is shown that the use of the block approach and S-shaped smooth and bound feedback [28,29,30,31] makes it possible to consider the constraints on the velocity and acceleration of the control plant. Therefore, generator variables produce a naturally smoothed space curve and its derivatives in real time. Hence, these variables are used to form the control law in the UAV. We present the simulation results, which demonstrate the fulfillment of the design constraints in a closed-loop tracking system with a dynamic generator. Section 3.3 additionally considers the aspects of filtration of reference actions. We put forward various hypotheses about filtration, which are confirmed by numerical simulation. Finally, we present the results of a comparative analysis of closed-loop tracking systems with a dynamic generator and with various cases for installing additional low-pass filters with noisy reference actions.

## 2. Problem Definition

For an unmanned aerial vehicle (UAV) of an aircraft type, let us consider the problem of automatic control of movement along a given path. It is assumed that the UAV has a rigid body with an axis of symmetry, the Earth is flat and motionless; the atmosphere is at rest relative to the Earth. We consider the model of the spatial movement of the center of mass (material point) of the UAV in the trajectory coordinate system [32] as a mathematical model of the control plant:

(1) $$\begin{array}{l} \dot{L}=V\cos\theta \cos\Psi ,\;\;\dot{H}=V\sin\theta ,\;\;\dot{Z}=-V\cos\theta \sin\Psi ;\\ \dot{V}=(n_{x}-\sin\theta )g,\;\;\dot{\theta }=\frac{(n_{y}\cos\gamma -\cos\theta )g}{V},\;\;\dot{\Psi }=-\frac{gn_{y}\sin\gamma }{V\cos\theta }, \end{array}$$

where L is the longitudinal flight range, H is the flight altitude, Z is the lateral deviation, V is the ground velocity, θ is the angle of inclination of the flight path to the horizon, Ψ is the flight path angle, and g=9.8 $\left[m/s^{2}\right]$ is the gravitational acceleration. As a control $u={\left(u_{1},\;u_{2},\;u_{3}\right)}^{T}$, we take the longitudinal $n_{x}$ and transverse $n_{y}$ overloads and the roll angle γ, $\left|\gamma \right|<\pi$ in the following form:

(2) $$u_{1}=n_{x},\;u_{2}=n_{y}\cos\gamma ,\;u_{3}=n_{y}\sin\gamma .$$

The output (controlled) variables of the model (1) are spatial coordinates that determine the position of the center of mass of the UAV in the flight mode in the trajectory coordinate system. Let us introduce the following notation for the vector of controlled variables and their velocities:

(3) $$y_{1}={\left(y_{11}:=L,\;y_{12}:=H,\;y_{13}:=Z\right)}^{T},\;{\dot{y}}_{1}=y_{2}={\left(y_{21}:=\dot{L},\;y_{22}:=\dot{H},\;y_{23}:=\dot{Z}\right)}^{T}.$$

The standard problem is to use control as a function of a time or in the form of feedback to ensure that the output variables $y_{1}(t)$ track the reference actions $\chi_{1}(t)={\left(\chi_{11}(t),\;\chi_{12}(t),\;\;\chi_{13}(t)\right)}^{T}$, which determine the desired path. Let us assume that there are no wind disturbances, and all state variables are measured. The reference actions are realizable by the given UAV, and their first and second derivatives are known. Under these conditions, we can provide asymptotic stabilization of tracking errors $\xi_{1}(t)={\left(\xi_{11}(t),\;\xi_{12}(t),\;\xi_{13}(t)\right)}^{T}$, namely,

(4) $$\underset{t\to +\infty }{\lim}\xi_{1j}(t)=0,\;\xi_{1j}(t)=y_{1j}(t)-\chi_{1j}(t),\;j=1,\;2,\;3.$$

To solve problem (4), we apply a typical feedback linearization technique, which is convenient for designing a tracking system in nonlinear minimum-phase control plants [33]. The synthesis procedure consists of three stages.

In the first stage, we transform the mathematical model of control plant (1)–(2) to the input–output form in the coordinate basis of variables (3). In the flight mode, the conditions V>0, $\left|\theta (t)\right|<\pi /2,$ $\left|\Psi (t)\right|<\pi /2,$ t≥0 are met. Therefore, there are diffeomorphic changes of variables,

(5) $$y_{21}:=V\cos\theta \cos\Psi ,\;\;\;y_{22}:=V\sin\theta ,\;\;\;y_{23}:=-V\cos\theta \sin\Psi ,$$

which transform (1)–(2) to the canonical form,

(6) $$\begin{array}{l} {\dot{y}}_{1}=y_{2},\;\;\\ {\dot{y}}_{2}=ag+B(\theta ,\;\Psi )gu, \end{array}$$

where $a={\left(0;\;\;-1;\;\;0\right)}^{T};$

$$B=\left(\begin{array}{ccc} \cos\theta \cos\Psi & -\sin\theta \cos\Psi & \sin\Psi \\ \sin\theta & \cos\theta & 0\\ -\cos\theta \sin\Psi & \sin\theta \sin\Psi & \cos\Psi \end{array}\right),\;\det B\equiv 1,\;B^{-1}=B^{T},$$

(7) $$\begin{array}{l} V(t)=\sqrt{y_{21}^{2}(t)+y_{22}^{2}(t)+y_{23}^{2}(t)},\;y_{21}^{2}(t)+y_{23}^{2}(t)\neq 0,\;\\ \sin\theta (t)=\frac{y_{22}(t)}{V(t)},\;\cos\theta =\sqrt{1-\frac{y_{22}^{2}(t)}{V^{2}(t)}},\;\\ \cos\Psi (t)=\frac{y_{21}(t)}{V(t)\cos\theta (t)},\;\sin\Psi (t)=-\frac{y_{23}(t)}{V(t)\cos\theta (t)}. \end{array}$$

In the second stage, we will represent system (6) on the coordinate basis of tracking errors $\xi_{1}=y_{1}-\chi_{1}$ and their derivatives ${\dot{\xi }}_{1}=\xi_{2}={\dot{y}}_{1}-{\dot{\chi }}_{1}$, namely,

(8) $$\begin{array}{l} {\dot{\xi }}_{1}=\xi_{2},\;\;\\ {\dot{\xi }}_{2}=ag-{\ddot{\chi }}_{1}+B(\theta ,\;\Psi )gu. \end{array}$$

In the third stage, we will form the control law in the form of feedback,

(9) $$\begin{array}{l} u=B^{T}(\theta ,\;\Psi )(-C_{1}\xi_{1}-C_{2}\xi_{2}-ag+{\ddot{\chi }}_{1})/g=\\ =-B^{T}(\theta ,\;\Psi )(C_{1}(y_{1}-\chi_{1})+C_{2}(y_{2}-{\dot{\chi }}_{1})+ag-{\ddot{\chi }}_{1})/g,\\ C_{i}=\mathrm{diag}\{c_{ij}\},\;c_{ij}=\mathrm{const}>0,\;i=1,\;2,\;j=1,\;2,\;3, \end{array}$$

which linearizes the closed-loop virtual system (8),

$${\dot{\xi }}_{1}=\xi_{2},\;{\dot{\xi }}_{2}=-C_{1}\xi_{1}-C_{2}\xi_{2}$$

and provides its matrix with the desired eigenvalues $\lambda_{1j},\;\lambda_{2j},$ Re(λij)<0: $c_{1j}=\lambda_{1j}\lambda_{2j},$ $c_{2j}=-\lambda_{1j}-\lambda_{2j},$ j=1, 2, 3. The goal of control is achieved both in the given system and in the closed-loop system (6), (9) accordingly:

(10) $$\begin{array}{l} {\dot{y}}_{1}=y_{2},\;\;\\ {\dot{y}}_{2}=-C_{1}y_{1}-C_{2}y_{2}+C_{1}\chi_{1}+C_{2}{\dot{\chi }}_{1}+{\ddot{\chi }}_{1}. \end{array}$$

Note that the control law as a function of a time that provides (4) has a form similar to (9), in the sense that its implementation requires knowing the first ${\dot{\chi }}_{1}(t)$ and the second ${\ddot{\chi }}_{1}(t)$ derivatives of the vector reference action.

In this paper, we consider the problem related to the features of the reference actions $\chi_{1}(t)$. Let us introduce the following assumptions.

1. Vector signal $\chi_{1}(t)={\left(\chi_{11}(t),\;\chi_{12}(t),\;\;\chi_{13}(t)\right)}^{T}$ enters the control system in real time from an autonomous source, its analytical form and its derivatives are not known in advance. The first problem follows from this; it is necessary to restore the first and second derivatives of this signal in real time to form the control law (9).
2. The unknown noises $\eta (t)={\left(\eta_{1}(t),\;\eta_{2}(t),\;\;\eta_{3}(t)\right)}^{T}$ can be superimposed on the useful signal $\chi_{1}(t)$. Next, noisy vector signal ${\bar{\chi }}_{1}(t)=\chi_{1}(t)+\eta (t)$ enters the control system. It leads to the second problem associated with the need to filter the reference actions: it is necessary to isolate the useful signal and obtain its derivatives.
3. The components of the useful signal $\chi_{1j}(t),\;j=1,\;2,\;3$ are continuous. However, they are non-smooth functions of time (derivatives ${\dot{\chi }}_{1j}(t)$ have finite discontinuities). For example, such a situation is possible in the case when the specified path is constructed at the first approximation in the form of a spatial polyline connecting the reference waypoints. Hence, the third problem arises; it is necessary to smooth out the polyline junctions in real time to prevent outliers of the values of derivatives at discontinuity points. Otherwise, this will lead to unacceptable outliers of control actions. Additionally, the curvature of the approximating path must satisfy the constraints on the velocity and acceleration of a particular UAV in order for the reference action to be realizable.

As noted in the introduction, dynamic differentiators of one type or another can be used to solve the first two problems [4,5,6,7,8,9,10,11,12,13,14,15,16]. Let us briefly outline the principles of their construction. A dynamic differentiator with a filtering effect is constructed as a state observer of a virtual dynamic model of a useful signal, which has a canonical form. This model is required to restore the first and second derivatives of a vector signal $\chi_{1}(t)\in R^{3}$. It consists of three connected blocks of the third order, namely,

(11) $${\dot{\chi }}_{1}=\chi_{2},\;{\dot{\chi }}_{2}=\chi_{3},\;{\dot{\chi }}_{3}=\chi_{4},\;\chi_{i}\in R^{3},\;i=\bar{1,\;4}.$$

In system (11), the state variables are the useful signal and its first and second derivatives $\;{\dot{\chi }}_{1}=\chi_{2},\;{\ddot{\chi }}_{1}=\chi_{3}$. The third derivative ${\dddot{\chi }}_{1}=\chi_{4}$ is treated as an unknown bounded input. The state observer is constructed as a copy of model (11) in the form

(12) $${\dot{z}}_{1}=z_{2}+v_{1}({\bar{\chi }}_{1}-z_{1}),\;{\dot{z}}_{2}=z_{3}+v_{2}({\bar{\chi }}_{1}-z_{1}),\;{\dot{z}}_{3}=v_{3}({\bar{\chi }}_{1}-z_{1}),\;\;z_{i},\;v_{i}\in R^{3},\;\;i=\bar{1,\;3}.$$

Each i-th block has its own corrective action $v_{i}$, which is formed by the measured noisy signal ${\bar{\chi }}_{1}(t)=\chi_{1}(t)+\eta (t)$. The observation problem is reduced to the stabilization of observation errors $\varepsilon_{i}=\chi_{i}-z_{i}\in R^{3}$ in a virtual system with an undefined input

(13) $${\dot{\varepsilon }}_{1}=\varepsilon_{2}+v_{1}({\bar{\chi }}_{1}-z_{1}),\;{\dot{\varepsilon }}_{2}=\varepsilon_{3}+v_{2}({\bar{\chi }}_{1}-z_{1}),\;{\dot{\varepsilon }}_{3}=\chi_{4}+v_{3}({\bar{\chi }}_{1}-z_{1}).$$

The presence of an undefined input $\chi_{4}$ does not allow the use the standard Kalman linear correction [1]. In order to suppress disturbances in systems (12), (13), one can select discontinuous corrective actions [11]. Another approach is to expand the state space of the virtual system (11) by adding a low-pass filter, the input of which is a noisy signal [4]. The filter variables are used to form corrective actions of the extended observer, which improves the filtering properties of the algorithm.

Note that these methods are mainly used to process smooth noisy signals. Tuning of corrective actions is made so that the observer’s evaluation signals reproduce the useful signal and its derivatives as accurately as possible $z_{i}(t)\approx \chi_{i}(t),\;i=1,\;2,\;3$. Therefore, dynamic differentiators are not suitable for solving the third problem of smoothing reference signals and generating realizable spatial paths. By tuning these differentiators, it is rather difficult to consider the constraints on the state variables of the control plant.

The next section presents the main result. We propose a new approach to a comprehensive solution of all three of these problems and introduce a dynamic generator of realizable reference actions. It simultaneously filters and smooths the reference signal and restores its first and second derivatives.

## 3. Results

### 3.1. Substantiation of the Model and Design of a Dynamic Generator

As for the dynamic differentiator, we use the canonical model to construct a dynamic generator since one of its functions is the derivative restoring. In order to consider the properties of the control plant, let us use canonical system (6) as a basis for the construction. Moreover, there are constraints on the velocity and acceleration (overload) of a particular UAV, namely,

(14) $$\left\|y_{2}\right\|\leq \bar{V},\;\left\|u\right\|\leq U,\;\left\|\dot{u}\right\|\leq \bar{U},\;{\bar{V}}^{2}<U,\;U^{2}<\bar{U},$$

here and below $\left\|*\right\|$ is $l_{\infty }$-norm of the vector, $\bar{V},\;U,\;\bar{U}$ are design bounds on the norm for velocity, overload and the rate of change of overload for a particular UAV. The upper estimate of the acceleration vector norm is calculated using the value of U:

(15) $$\left\|{\dot{y}}_{2}\right\|\;=g\left\|a+B(\theta ,\;\Psi )u\right\|\leq 3gU.$$

The basic mathematical model of a dynamic generator for pre-processing of the reference action consists of two connected blocks of the third order,

(16) $${\dot{x}}_{1}=x_{2},\;{\dot{x}}_{2}=ag+Bgw.$$

The generator variables (16) have the following meaning: $w={\left(w_{1},\;w_{2},\;w_{3}\right)}^{T}$ is the vector of corrective actions. It must be selected in the form of feedback to ensure that the output variables of the generator $x_{1}(t)\in R^{3}$ track useful vector signal $\chi_{1}(t)\in R^{3}$. Next, $x_{1}={\left(x_{11},\;x_{12},\;x_{13}\right)}^{T}$ is estimation of the vector of reference actions, $x_{2}={\left(x_{21},\;x_{22},\;x_{23}\right)}^{T}$ and ${\dot{x}}_{2}={\left({\dot{x}}_{21},\;{\dot{x}}_{22},\;{\dot{x}}_{23}\right)}^{T}$ are restored first and second derivatives of reference actions, respectively. Thus, it is possible to solve the first posed problem due to the virtual system (16).

As was mentioned in Section 2, the useful signal elements are assumed to be piecewise-continuous deterministic functions of time with bounded derivatives. The smoothness requirements are not imposed on them, their analytical description is unknown. The constraints are understood as one-sided at the points of finite discontinuity. We assume that the norm of the vector of reference action velocity is less than the design bound on the norm of the UAV velocity vector in the areas of continuity

(17) $$\;\left\|{\dot{\chi }}_{1}(t)\right\|\leq X_{2}<\bar{V},\;t\geq 0.$$

Note that the dynamic generator (16) (as well as the dynamic differentiator (11)) is constructed autonomously from the control plant (1) and does not receive any signals from it. The feedback is formed according to the variables of system (16) and the reference action signal. However, vector variables $x_{2},\;w,\;\dot{w}$ of system (16) are analogs of the variables $y_{2},\;u,\;\dot{u}$ of the canonical model (6), respectively. Thus, we must provide the corresponding constraints (14), (15) on state variables and corrective actions in the closed-loop system (16), namely,

(18) $$\left\|x_{2}(t)\right\|\leq \bar{V},\;\;\left\|w(t)\right\|\leq U\Rightarrow \;\left\|{\dot{x}}_{2}(t)\right\|\leq 3gU,\;\;\left\|\dot{w}(t)\right\|\leq \bar{U},t\geq 0.$$

The fundamental difference in systems (12)–(13) and (16) tuning is that in system (16) we do not set the tracking error stabilization accuracy $e_{1}(t)=x_{1}(t)-\chi_{1}(t)$ a priori. Its minimum value is determined by constraints (18) and the response of controlled variables during the processing of jumps. As a result, the output of the generator $x_{1}(t)$ will produce a path that is a smoothed analog of the useful vector signal $\chi_{1}(t)$. Next, $x_{1}(t)$ enters control system as a reference action. It will be realizable by the plant since smoothing is produced naturally using dynamic model (16) considering the constraints on particular UAV. Thus, due to the dynamic generator (16), we can automatically provide an on-line solution to the third posed problem without complicated off-line geometric calculations (see, for example, [25]).

In order to fulfill the given constraints (18), we propose the use the block control principle in the synthesis of corrective actions w and nonlinear bounded feedback. Such feedback is S-shaped sigmoid function, which is a modification of the hyperbolic tangent σ(x)=−th(−x/2) [28,29,30,31]. This function is defined on the whole number line. It is a smooth, odd and bounded $\left|\sigma (x)\right|<1$. In a small neighborhood of zero $\left|x\right|\leq \Delta$, sigmoid function is similar to a linear function. While when $\left|x\right|>\Delta$ at infinity, it tends to sign function. We can say that the sigmoid function is a smooth analog of a linear function with saturation. Therefore, it can be used both as a fictitious and as a true control for stabilizing the controlled variables and suppressing matched external disturbances while meeting the requirements for smoothness and boundedness of control actions.

In order to tune the sigmoid feedback, we introduced two scaling factors into the sigmoid function k, m=const>0, namely,

$$m\sigma (kx)=m\frac{1-\exp(-kx)}{1+\exp(-kx)},$$

where a factor k determines the slope of the sigmoid function in a small neighborhood of zero. It plays a role as a high-gain factor in further constructions, and the tracking accuracy depends on its value. A factor m, which we will call the amplitude, determines the expansion of the sigmoid function along the vertical axis and limits its maximum in absolute value.

The derivative of the sigmoid function has a recursive form:

$$m\sigma^{\prime }(kx)=0.5mk(1-\sigma^{2}(kx)),\;\;0<m\sigma^{\prime }(kx)\leq 0.5mk,\;\;x\in R.$$

For the convenience of analyzing systems with sigmoid control, we introduce the points of division of the sigmoid function into conditionally linear and conditionally constant, σ(±2.2)≈±0.8. We obtained the number 2.2 by the criterion of the minimum of the basic estimate of the modulus of the change rate of the sigmoid control in an elementary system with an external disturbance [30]. Next, outside a small neighborhood of zero $\left|x\right|\leq \Delta$, we have,

(19) $$0.8m\leq m\left|\sigma (kx)\right|<m,\;\left|x\right|>\Delta \geq 2.2/k.$$

First, we will consider the basic procedure for tuning a dynamic generator (16) with sigmoid corrective actions that provide a solution to the first and third problems in a deterministic formulation. After that, we will additionally study its filtering properties.

### 3.2. Synthesis of a Dynamic Generator in a Deterministic Formulation

#### 3.2.1. Theoretical Background

For system (16), we pose the problem of synthesizing corrective actions that ensure tracking with some accuracy of an external vector signal $\chi_{1}(t)\in R^{3}$. Its elements are assumed to be deterministic. In the general case, they are non-smooth functions of time with unknown derivatives. The purpose of control is to fulfill the given constraints on state variables, corrective actions and their derivatives (18) in a closed-loop system. To solve this problem, we will perform the following actions:

- Write down the system (16) with respect to the tracking error and form sigmoid local feedback and corrective actions;
- Formulate sufficient conditions for stabilization of the obtained system, under which the tracking problem will be solved with some accuracy;
- Make sure that the variables $x_{2}(t),\;{\dot{x}}_{2}(t)$ will be bounded when t≥0 in the closed-loop system (16);
- Compose inequalities for selecting the parameters of corrective actions, under which the given constraints (18) will be fulfilled in closed-loop system (16).
- The fundamental difference from the tracking system synthesis procedure (6), (9) is that we do not know the first and second derivatives of the external signal. Therefore, in the first equation of system (16), written with respect to tracking errors ${\dot{e}}_{1}=x_{2}-{\dot{\chi }}_{1},$ the first derivative of the external signal ${\dot{\chi }}_{1}(t)$ is treated as an external bounded disturbance (17). To ensure the stabilization of tracking errors with invariance to the disturbance, we will consider the vector state variable $x_{2}$ as fictitious control. Next, let us form sigmoid local feedback

$$x_{2}^{*}=-m_{1}\sigma (k_{1}e_{1}),\;\;\sigma (k_{1}e_{1})={\left(\sigma (k_{1}e_{11}),\;\sigma (k_{1}e_{12}),\;\sigma (k_{1}e_{13})\right)}^{T},\;\;m_{1},\;k_{1}=\mathrm{const}>0.$$

This leads to the problem of stabilizing the residual between the real and the selected fictitious control,

(20) $$e_{2}=x_{2}-x_{2}^{*}=x_{2}+m_{1}\sigma (k_{1}e_{1}).$$

In order to solve this, we rewrite system (16) with respect to residuals $e_{1},\;e_{2}$

(21) $$\begin{array}{l} {\dot{e}}_{1}=-m_{1}\sigma (k_{1}e_{1})+e_{2}-{\dot{\chi }}_{1},\\ {\dot{e}}_{2}=ag+0.5m_{1}k_{1}\Lambda_{1}(x_{2}-{\dot{\chi }}_{1})+Bgw, \end{array}$$

where,

(22) $$\Lambda_{1}=\mathrm{diag}(\Lambda_{1j}),\;\Lambda_{1j}=1-\sigma^{2}(k_{1}e_{1j})),\;0<\Lambda_{1j}\leq 1,\;j=1,\;2,\;3.$$

Note that, in contrast to systems (6), (8), (16), the resulting system (21) is non-canonical. Using corrective actions, we compensate all known terms in the second equation of the virtual system (21) and introduce stabilizing sigmoid feedback, which depends on residual (20),

(23) $$\begin{array}{l} w=-B^{T}(m_{2}\sigma (k_{2}e_{2})+ag+0.5m_{1}k_{1}\Lambda_{1}x_{2})/g,\\ m_{2},\;k_{2}=\mathrm{const}>0,\;\;\sigma (k_{2}e_{2})=(\sigma (k_{2}e_{21}),\;\sigma (k_{2}e_{22}),\;\sigma (k_{2}e_{23})){\;}^{T}.\; \end{array}$$

As a result, we obtain closed-loop virtual system (21), (23) of the form,

(24) $$\begin{array}{l} {\dot{e}}_{1}=-m_{1}\sigma (k_{1}e_{1})+e_{2}-{\dot{\chi }}_{1},\\ {\dot{e}}_{2}=-m_{2}\sigma (k_{2}e_{2})-0.5m_{1}k_{1}\Lambda_{1}{\dot{\chi }}_{1}. \end{array}$$

We must select amplitude values of sigmoid feedback $m_{1},\;m_{2}>0$ so that the variables of the virtual system (24) sequentially converge in the following neighborhoods of zero (19):

(25) $$\left\|e_{2}(t)\right\|\leq 2.2/k_{2}\Rightarrow \left\|e_{1}(t)\right\|\leq 2.2/k_{1},$$

where the values of high-gain factors $k_{1},\;k_{2}>0$ determine the accuracy of stabilization of the residuals in the steady state.

Let us use the second Lyapunov method and evaluate the terms of the derivatives of the quadratic form $0.5e_{1}^{T}e_{1}+0.5e_{2}^{T}e_{2}$ outside the neighborhoods (25). Using (19), (22), we do it as following:

(26) $$\begin{array}{l} e_{1}^{T}{\dot{e}}_{1}=e_{1}^{T}(-m_{1}\sigma (k_{1}e_{1})+e_{2}-{\dot{\chi }}_{1})\leq \left\|e_{1}\right\|(\left\|e_{2}\right\|+\left\|{\dot{\chi }}_{1}\right\|-0.8m_{1}),\\ e_{2}^{T}{\dot{e}}_{2}=e_{2}^{T}(-m_{2}\sigma (k_{2}e_{2})-0.5m_{1}k_{1}\Lambda_{1}{\dot{\chi }}_{1})\leq \left\|e_{2}\right\|(0.5m_{1}k_{1}\left\|{\dot{\chi }}_{1}\right\|-0.8m_{2}). \end{array}$$

Stabilization of residuals with some accuracy (25) will be provided if expressions (26) are negative. With respect (17), sufficient conditions are met for the following values of the amplitudes:

(27) $$\begin{array}{l} 0.8m_{2}>0.5m_{1}k_{1}X_{2}\Leftrightarrow m_{2}>0.625m_{1}k_{1}X_{2}\Rightarrow \left\|e_{2}(t)\right\|\leq 2.2/k_{2}\Rightarrow \\ 0.8m_{1}>2.2/k_{2}+X_{2}\Leftrightarrow m_{1}>2.75/k_{2}+1.25X_{2}\Rightarrow \left\|e_{1}(t)\right\|\leq 2.2/k_{1}. \end{array}$$

In the dynamic generator (16) (as well as in the dynamic differentiator (12)), we can set any initial values of the state variables. To ensure (25) and, therefore, $m_{i}\left|\sigma (k_{i}e_{ij})\right|\leq 0.8m_{i},\;i=1,\;2;\;j=1,\;2,\;3$ when t≥0, it is beneficial for us to set the following initial values,

(28) $$x_{1}(0)=\chi_{1}(0)\Rightarrow e_{1}(0)=\vec{0}\Rightarrow e_{2}(0)=x_{2}(0),\;0<\left\|x_{2}(0)\right\|<2.2/k_{2}.$$

It follows from (20) that $x_{2}=e_{2}-m_{1}\sigma (k_{1}e_{1}).$ This variable is an analog of velocity vector of UAV. Therefore, if we select amplitudes to fulfill (27), the norm of $x_{2}(t)$ will be bounded:

(29) $$\left\|x_{2}(t)\right\|\leq 2.2/k_{2}+0.8m_{1},\;\;t\geq 0.$$

We initially introduced corrective actions in (23) to justify the final form of the dynamic generator, which is implemented as a closed-loop system (16), (23) and does not depend on the matrix B(θ, Ψ):

(30) $$\begin{array}{l} {\dot{x}}_{1}=x_{2},\;\\ {\dot{x}}_{2}=-m_{2}\sigma (k_{2}e_{2})-0.5m_{1}k_{1}\Lambda_{1}x_{2}=-m_{2}\sigma (k_{2}(x_{2}+m_{1}\sigma (k_{1}(x_{1}-\chi_{1}))))-\\ -0.5m_{1}k_{1}\left(\begin{array}{ccc} 1-\sigma^{2}(k_{1}(x_{11}-\chi_{11})) & 0 & 0\\ 0 & 1-\sigma^{2}(k_{1}((x_{12}-\chi_{12}))) & 0\\ 0 & 0 & 1-\sigma^{2}(k_{1}((x_{13}-\chi_{13}))) \end{array}\right)x_{2}. \end{array}$$

The variable ${\dot{x}}_{2}(t)$ is analog to the UAV acceleration vector. Using (22) and (29), we can see that the norm of ${\dot{x}}_{2}(t)$ is also bounded in (30):

(31) $$\left\|{\dot{x}}_{2}(t)\right\|\leq 0.8m_{2}+0.5m_{1}k_{1}(2.2/k_{2}+0.8m_{1}),\;t\geq 0.$$

Note that the dynamic generator (30) contains fewer blocks than the standard dynamic differentiator (12). In system (30), the estimated signal of the second derivative of the reference signal ${\ddot{\chi }}_{1}(t)$ is the right side of the second block of the system (30). It is defined by the expression $-m_{2}\sigma (k_{2}e_{2}(t))-0.5m_{1}k_{1}\Lambda_{1}(t)x_{2}(t)$.

Using (27), (29) and (31), let us compose a system of double inequalities for selecting the parameters of the sigmoid feedback that satisfies the given constraints (18):

(32) $$\left\{\begin{array}{l} 2.2/k_{2}+X_{2}<0.8m_{1}\leq \bar{V}-2.2/k_{2}\Rightarrow k_{2}>4.4/(\bar{V}-X_{2}),\\ 0.5m_{1}k_{1}X_{2}<0.8m_{2}\leq 3gU-0.5m_{1}k_{1}\bar{V}\Rightarrow m_{1}k_{1}<6gU/(\bar{V}+X_{2}). \end{array}\right.$$

Double inequalities (32) are consistent and have a non-empty set of solutions due to a priori assumptions (14).

Let ${\bar{k}}_{2}(\bar{U})$ be the maximum allowable value of a high-gain factor $k_{2}$, determined by the design constraints on the rate of control change (14). We take a specific value $k_{2}=k_{2}^{*}$ under the assumption that the following condition,

(33) $$4.4/(\bar{V}-X_{2})<k_{2}^{*}\leq {\bar{k}}_{2}(\bar{U})$$

is met. Let us take a specific value of $m_{1}=m_{1}^{*}$ based on the first inequality (32), namely,

(34) $$2.75/k_{2}^{*}+1.25X_{2}<m_{1}^{*}\leq 1.25\bar{V}-2.75/k_{2}^{*}.$$

Finally, based on the second inequality (32), we successively take specific values $k_{1}=k_{1}^{*}$ and $m_{2}=m_{2}^{*}$:

(35) $$0<k_{1}^{*}<\frac{6gU}{m_{1}^{*}(\bar{V}+X_{2})};\;\;0.625m_{1}^{*}k_{1}^{*}X_{2}<m_{2}^{*}\leq 3.75gU-0.625m_{1}^{*}k_{1}^{*}\bar{V}.$$

Upper bounds for selection high-gain factors $k_{1}$ (35) and $k_{2}$ (33) determine the threshold values of the stabilization accuracy of the tracking error and residual (25),

(36) $$\begin{array}{l} \left\|e_{1}(t)\right\|\leq \Delta_{1},\;\Delta_{1}>\frac{0.04m_{1}^{*}(\bar{V}+X_{2})}{U}\geq \frac{0.04(2.75/{\bar{k}}_{2}(\bar{U})+1.25X_{2})(\bar{V}+X_{2})}{U};\;\;\\ \left\|e_{2}(t)\right\|\leq \Delta_{2},\;\Delta_{2}\geq \frac{2.2}{{\bar{k}}_{2}(\bar{U})}. \end{array}$$

It follows from estimates (36) that the following values should be set to minimize the tracking error: $k_{2}^{*}={\bar{k}}_{2}(\bar{U})-\alpha ,$ $m_{1}^{*}=2.75/k_{2}^{*}+1.25X_{2}+\alpha ,$ where α is positive small constant. Note that with the accepted initial values (28), there is no need to waste the resources of sigmoid feedback on acquiring the state variables of the virtual system (24) in the neighborhood of zero (25).

Thus, the variables of the dynamic generator (30) produce smoothed vector signals $x_{1}(t),$ $x_{2}(t),$ ${\dot{x}}_{2}(t)$ after processing the reference non-smooth signal $\chi_{1}(t)$. These signals are realizable for a particular UAV. They are used in real time in the control law (9) instead of $\chi_{1}(t),$ ${\dot{\chi }}_{1}(t),$ ${\ddot{\chi }}_{1}(t)$, namely,

(37) $$u=-B^{T}(C_{1}(y_{1}-x_{1})+C_{2}(y_{2}-x_{2})+ag-{\dot{x}}_{2})/g.$$

Additionally, they do not result in inadmissible overloads.

#### 3.2.2. Simulation Results

For numerical simulation of the developed algorithms (which was carried out in MATLAB-Simulink with the Euler integration method with a fixed step of 0.0001 s), we considered a micro-UAV, which parameters are summarized in Table 1. Such aircraft perform reconnaissance functions and provide information about the current situation.

The reference signal determines the desired path of the UAV at the first approximation. It is given in the form of a continuous but non-smooth spatial broken line:

(38) $$\begin{array}{l} \chi_{11}=0,\;\chi_{12}=2t+100,\;\chi_{13}=2t,\;\;\;t\in [0;\;5);\\ \chi_{11}=2t-10,\;\chi_{12}=115-t,\;\chi_{13}=10,\;\;\;t\in [5;\;10);\\ \chi_{11}=10,\;\chi_{12}=125-2t,\;\chi_{13}=30-2t,\;\;\;t\in [10;\;15);\\ \chi_{11}=40-2t,\;\chi_{12}=t+80,\;\chi_{13}=0,\;\;\;t\in [15;\;20);\\ \chi_{11}=0,\;\chi_{12}=2t+60,\;\chi_{13}=2t-40,\;\;\;t\geq 20,\;\;\chi_{1j}\;[m],\;\;t\;[s]. \end{array}$$

For this plant, a dynamic generator (30) was constructed with the initial values $x_{1}(0)=\chi_{1}\left(0\right)=\left(0,\;100,\;0\right){\;}^{T},$ $x_{2}(0)={\left(0.01,\;\;0.01,\;\;0.01\right)}^{T}$ and with the parameters, accepted on the basis of inequalities (32)–(35) with respect to the constraints of this UAV on velocity and overload in the form:

(39) $$m_{1}=4,\;\;k_{1}=4\;,\;\;m_{2}=22,\;\;k_{2}=10.$$

**Figure 1:**
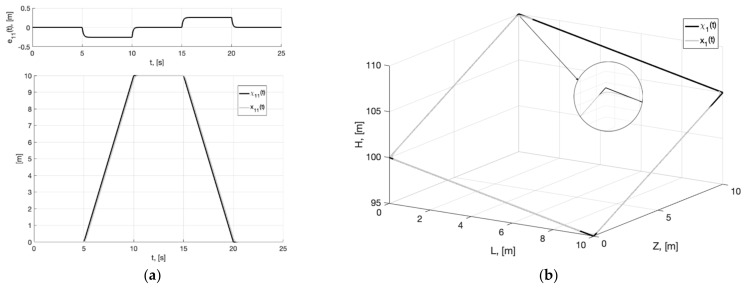
In (**a**), plots of the reference signal $\chi_{11}(t)$ (38) and its processing of $x_{11}(t)$ (shown below) and tracking error $e_{11}(t)=x_{11}(t)-\chi_{11}(t)$ (shown above). In (**b**), spatial plots of the reference polyline $\chi_{1}(t)$ and point in phase space $x_{1}(t)$ of dynamic generator (30).

**Figure 2:**
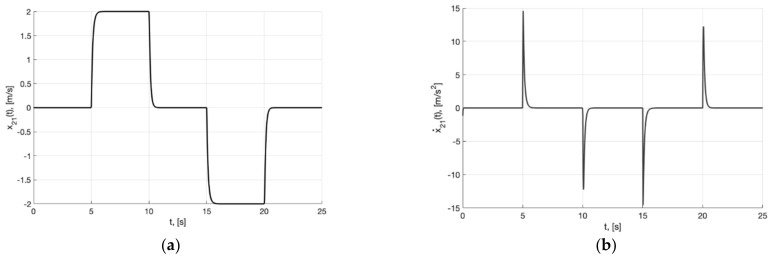
In (**a**), plot of $x_{21}(t).$ In (**b**), plot of ${\dot{x}}_{21}=-m_{2}\sigma (k_{2}e_{21})-0.5m_{1}k_{1}\Lambda_{11}x_{21}$ of generator (30).

In Figure 1a, we present the plots of the first component of the reference signal $\chi_{11}(t)$ (38), its processing of the corresponding output variable $x_{11}(t)$ of generator (30), and tracking error $e_{11}(t)=x_{11}(t)-\chi_{11}(t).$ For the rest of the components of vectors $\chi_{1}(t)$, $x_{1}(t)$, $e_{1}(t)=x_{1}(t)-\chi_{1}(t)$, the graphical results are similar. We can see from Figure 2a that $\left|e_{11}(t)\right|\leq 0.256$ m. In Figure 1b, we show the spatial plots of the reference polyline $\chi_{1}(t)=(\chi_{11}(t),\;\chi_{12}(t),\;\;\chi_{13}(t))$ and output variable $x_{1}(t)=(x_{11}(t),\;x_{12}(t),\;\;x_{13}(t))$ of generator (30). We see the smoothing of sharp corners at singular points.

Figure 2a,b present the plots of $x_{21}(t)$ and ${\dot{x}}_{21}=-m_{2}\sigma (k_{2}e_{21})-0.5m_{1}k_{1}\Lambda_{11}x_{21},$ which describe the processing of the first and second derivative of the first component of the reference signal ${\dot{\chi }}_{11}(t)$ and ${\ddot{\chi }}_{11}(t)$ by generator, respectively.

As was noted, the generator variables enter the UAV control system as reference actions and their first and second derivatives. In the control law (37), we accepted the following feedback parameters:

$$C_{i}=\mathrm{diag}\{14,\;15.5,\;14.5\},\;C_{2}=\mathrm{diag}\{167,\;166,\;165\}.$$

**Figure 3:**
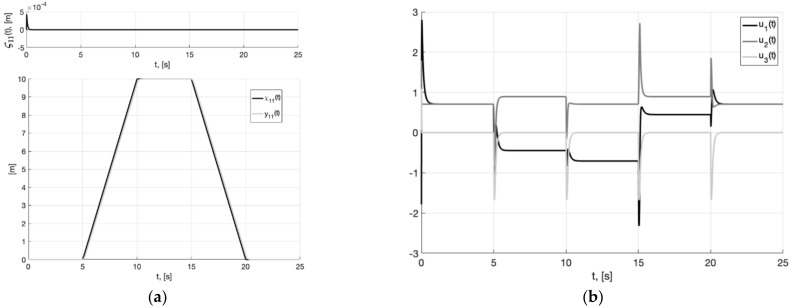
In (**a**), plots of the reference signal $\chi_{11}(t)$ (38), output variable $y_{11}(t)$ of closed-loop system (6), (37) with generator (30) (shown below) and tracking error $\varsigma_{11}(t)=y_{11}(t)-x_{11}(t)$ (shown above). In (**b**), plots of the control actions $u_{j}(t),\;j=1,\;2,\;3$ (37).

In Figure 3a, we show the reference signal $\chi_{11}(t)$ (38), and output variable $y_{11}(t)$ of closed-loop system (6), (37) with generator (30), tracking error $\varsigma_{11}(t)=y_{11}(t)-x_{11}(t)$, arising when the plant tracks the smooth output signal of the generator $x_{11}(t)$. It is a realizable signal. Therefore, asymptotic stabilization of the tracking error is ensured in a closed-loop system. In Figure 3b, we show plots of control actions $u_{1}(t),\;u_{2}(t),\;u_{3}(t)$ (37). They are bounded, their values correspond to permissible overloads (2).

### 3.3. Filtering Methods

#### 3.3.1. Research Hypotheses

Consider the case when an unknown noise $\eta (t)={\left(\eta_{1}(t),\;\eta_{2}(t),\;\;\eta_{3}(t)\right)}^{T}$ is superimposed on the useful signal $\chi_{1}(t)={\left(\chi_{11}(t),\;\chi_{12}(t),\;\;\chi_{13}(t)\right)}^{T}$. Here, the noisy signal ${\bar{\chi }}_{1}(t)=\chi_{1}(t)+\eta (t)$ enters the control system. Let us assume that η(t) is normal random variable with zero mean and bounded variance. Next, the basic generator model (30) has the following form:

(40) $$\begin{array}{l} {\dot{x}}_{1}=x_{2},\;\\ {\dot{x}}_{2}=-m_{2}\sigma (k_{2}(x_{2}+m_{1}\sigma (k_{1}(x_{1}-{\bar{\chi }}_{1}))))-0,5m_{1}k_{1}\mathrm{diag}\{1-\sigma^{2}(k_{1}(x_{1j}-{\bar{\chi }}_{1j}))\}x_{2}. \end{array}$$

We introduce the following hypotheses based on a comparative analysis of the dynamic generator (40) and the dynamic differentiator (12). The differentiator (12) solves the observation problem. Due to this method, the noisy signal ${\bar{\chi }}_{1}(t)$ is present in all equations of the system (12). Generator (40) solves the tracking problem. According to this method, it is a block integrator of the second order, where the noisy signal acts only on the input in the form of arguments of sigmoid functions. In such a system, the noise at the input has little effect on the output due to natural filtering by integrators. The parasitic components are present in vector signals $x_{1}(t),\;x_{2}(t)$, which restore the reference signal and its first derivative, respectively. Thus, it should be expected that these parasitic components will be small enough without installing additional filters. The closed-loop system (6), (37) is also a block integrator of the second order, in which the noise together with the signal ${\dot{x}}_{2}(t)$ act only on the input. With respect (40), expressions (37), (10) take the form, respectively:

(41) $$\begin{array}{l} u=-B^{T}(C_{1}(y_{1}-x_{1})+C_{2}(y_{2}-x_{2})+ag+m_{2}\sigma (k_{2}(x_{2}+m_{1}\sigma (k_{1}(x_{1}-{\bar{\chi }}_{1}))))+\\ +0.5m_{1}k_{1}\mathrm{diag}\{1-\sigma^{2}(k_{1}(x_{1j}-{\bar{\chi }}_{1j}))\}x_{2})/g; \end{array}$$

(42) $$\begin{array}{l} {\dot{y}}_{1}=y_{2},\;\;\\ {\dot{y}}_{2}=-C_{1}y_{1}-C_{2}y_{2}+C_{1}x_{1}+C_{2}x_{2}-m_{2}\sigma (k_{2}(x_{2}+m_{1}\sigma (k_{1}(x_{1}-{\bar{\chi }}_{1}))))-\\ -0.5m_{1}k_{1}\mathrm{diag}\{1-\sigma^{2}(k_{1}(x_{1j}-{\bar{\chi }}_{1j}))\}x_{2}. \end{array}$$

The first hypothesis is that, in the closed-loop system (42), it is possible to ensure the invariance of the output $y_{1}(t)$ to the noise in the reference action without additional filters.

At the input of system (40) and, consequently, system (42), parasitic noises are present in the form of arguments of sigmoid functions with gain factor $k_{1}(x_{1}-{\bar{\chi }}_{1})$. The second hypothesis is that one should also reduce the value of gain factor $k_{1}$ for better filtering. However, its reduction leads to an increase in the modulus of tracking errors $e_{1}(t)=x_{1}(t)-\chi_{1}(t)$ and, therefore, $\xi_{1}=y_{1}-\chi_{1}$ in the steady state. This is an analog of the well-known Kalman filter problem: “the higher the convergence rate of errors, the worse the filtering and vice versa.” To solve the problem, a compromise is established between the convergence rate and filtering properties. It is provided with a solution to the optimization problem.

Note that, in this paper, we do not consider the dynamics of the actuators in the model of the control plant (1). In the general UAV control system, in which Equation (1) are a top-level subsystem, overloads (2) perform a dual function. In Equation (1), they are control actions, and for UAV actuators, they can be interpreted as reference actions. Therefore, one cannot ignore the problem of filtering.

To provide filtering ${\dot{x}}_{2}(t)$ (40) and hence u(t) (41), it is necessary to introduce additional low-frequency filters on the reference signal ${\bar{\chi }}_{1}(t)$. The filter is added on ${\bar{\chi }}_{1}(t)$ before entering the generator (40) and/or on the second derivative of generator ${\dot{x}}_{2}(t)$ before it enters the control law (37).

The third hypothesis is that the filtering problem ${\dot{x}}_{2}(t)$ and u(t) can be solved naturally if we increase the dynamic order of generator (40). For this, one should take the extended system instead of system (6) as a basis for generator construction:

$${\dot{y}}_{1}=y_{2},\;\;{\dot{y}}_{2}=y_{3},\;\;{\dot{y}}_{3}=\frac{d}{dt}(B(\theta ,\;\Psi )gu)$$

or, continuing differentiation, a canonical system consisting of four blocks. The dynamic generator is constructed as a copy of such an extended model. The procedure for its synthesizing will be similar to the procedure outlined in Section 3.2.1. Therefore, the noisy signal will also enter only to the input of the extended generator. However, in contrast with the generator (40), the estimated signal of the second derivative of the reference signal will be formed not from the input, but from the output of the integrating block element. Additionally, in an extended generator consisting of four blocks, it will be separated from the noisy input by another block integrator. It will provide natural filtering and significantly reduce the impact of parasitic noise on the estimation of the second derivative and, consequently, on control (37).

The verification of this hypothesis requires additional, rather cumbersome calculation. It is not considered in this paper. For simplicity of presentation, we restrict ourselves to testing the first two hypotheses using numerical simulation. We also consider various cases for installing additional low-pass filters to improve the quality of vector signals ${\dot{x}}_{2}(t)$ and u(t).

#### 3.3.2. Simulation Results

In this section, we present the results of simulation the closed-loop system (6), (37), (40) in the following configurations:

1. Experiment 1: no additional filters;
2. Experiment 2: with additional low-pass filters for the reference signal ${\bar{\chi }}_{1}(t)$ before it enters generator (40);
3. Experiment 3: with additional low-pass filters on the second derivative ${\dot{x}}_{2}(t)$ of the generator before it enters control law (37);
4. Experiment 4: with additional low-pass filters for a reference signal ${\bar{\chi }}_{1}(t)$ and for the second derivative ${\dot{x}}_{2}(t)$ of generator.

In all these experiments, the reference action has the form,

$${\bar{\chi }}_{1}(t)=\chi_{1}(t)+\eta (t),$$

where the useful signal $\chi_{1}(t)$ is determined by (38), η(t) is a normal random variable with zero mean and variance 0.1. It is generated using the Simulink block “Random Number”.

For all experiments, we present the graphs similar to those on Figure 1, Figure 2 and Figure 3. Here, for comparison with the output signals of the generator and the control plant (on graphs similar to those on Figure 1 and Figure 3a), we show not a noisy signal ${\bar{\chi }}_{1}(t)$, but its useful component $\chi_{1}(t)$ (38). At the end of the section, we present the results of a comparative analysis of the conducted experiments.

**Figure 4:**
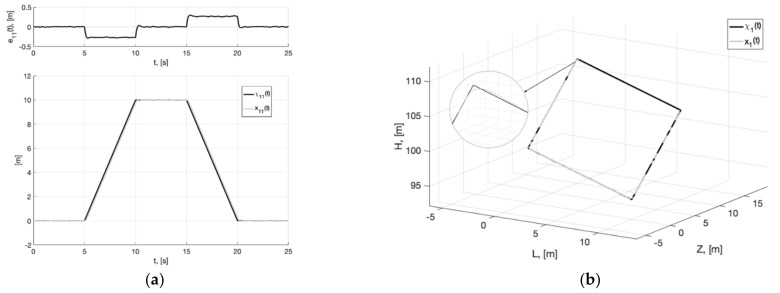
Experiment 1a. In (**a**), plots of the reference signal $\chi_{11}(t)$ (38) and its processing of $x_{11}(t)$ (shown below) and tracking error $e_{11}(t)=x_{11}(t)-\chi_{11}(t)$ (shown above). In (**b**), spatial plots of the reference polyline $\chi_{1}(t)$ and point in phase space $x_{1}(t)$ of dynamic generator (40).

**Figure 5:**
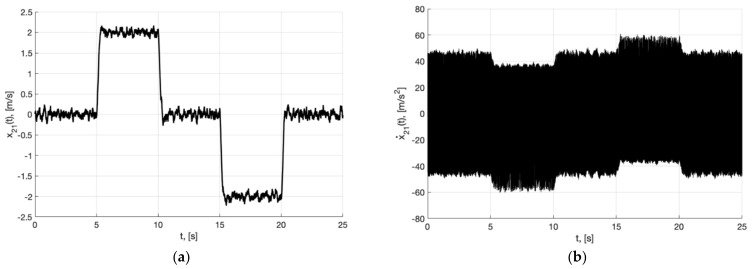
Experiment 1a. In (**a**), plot of $x_{21}(t).$ In (**b**), plot of ${\dot{x}}_{21}(t)$ of generator (40).

**Figure 6:**
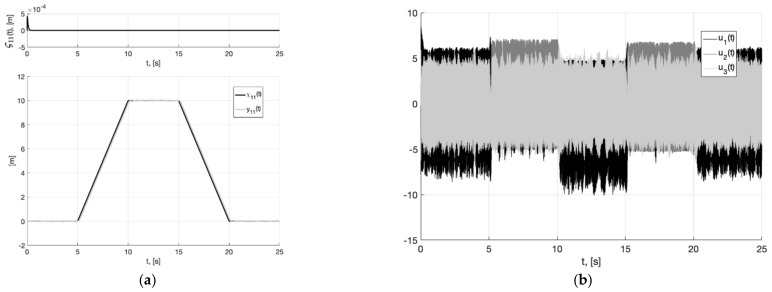
Experiment 1a. In (**a**), plots of the reference signal $\chi_{11}(t)$ (38) and output variable $y_{11}(t)$ of closed-loop system (42) (shown below) and tracking error $\varsigma_{11}(t)=y_{11}(t)-x_{11}(t)$ (shown above). In (**b**), plots of the control actions $u_{j}(t),\;j=1,\;2,\;3$ (41).

Figure 4, Figure 5 and Figure 6 show the graphs of Experiment 1a: the results of simulation the system (6), (37), (40) without additional filters with generator parameters (39).

**Figure 7:**
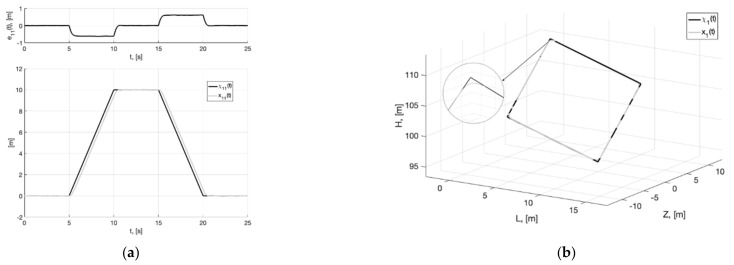
Experiment 1b. In (**a**), plots of the reference signal $\chi_{11}(t)$ (38) and its processing of $x_{11}(t)$ (shown below) and tracking error $e_{11}(t)=x_{11}(t)-\chi_{11}(t)$ (shown above). In (**b**), spatial plots of the reference polyline $\chi_{1}(t)$ and point in phase space $x_{1}(t)$ of dynamic generator (40).

**Figure 8:**
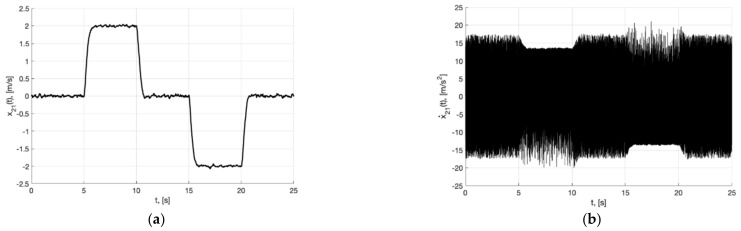
Experiment 1b. In (**a**), plot of $x_{21}(t).$ In (**b**), plot of ${\dot{x}}_{21}(t)$ of generator (40).

**Figure 9:**
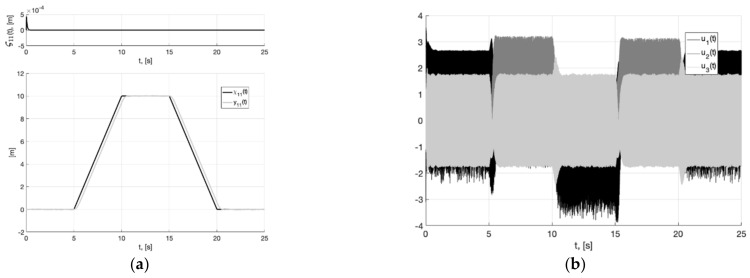
Experiment 1b. In (**a**), plots of the reference signal $\chi_{11}(t)$ (38) and output variable $y_{11}(t)$ of closed-loop system (42) (shown below) and tracking error $\varsigma_{11}(t)=y_{11}(t)-x_{11}(t)$ (shown above). In (**b**), plots of the control actions $u_{j}(t),\;j=1,\;2,\;3$ (41).

Figure 7, Figure 8 and Figure 9 show the graphs of Experiment 1b: the results of simulation the system (6), (37), (40) without additional filters with generator parameters,

(43) $$m_{1}=4,\;\;k_{1}=1.5\;,\;\;m_{2}=8,\;\;k_{2}=10,$$

where the value of gain factor $k_{1}$ is 2.6 times less than in Experiment 1a (39).

As can be seen from Figure 4, Figure 5, Figure 6, Figure 7, Figure 8 and Figure 9, the first and second hypotheses are confirmed. In the signals $x_{21}(t)\approx {\dot{\chi }}_{21}(t)$ (Figure 5a and Figure 8a) and especially in $x_{1}(t)\approx \chi_{1}(t)$ (Figure 4 and Figure 7), the parasitic component is quite small. The effects of corner smoothing, boundness of all variables and controls (Figure 6b and Figure 9b) are preserved. The output signals of closed-loop systems (Figure 6a and Figure 9a) are very similar to the output signals of a closed-loop system with a noisy reference (Figure 3a). In Experiment 1b (Figure 7, Figure 8 and Figure 9) the noisy of signal is less compared to Experiment 1a (Figure 4, Figure 5 and Figure 6). This is achieved by reducing the value of gain factor $k_{1}$ (41) by 2.6 times. However, we see (Figure 4a and Figure 7a) that the values of tracking errors are increased. In both cases, the signals ${\dot{x}}_{21}(t)$ (Figure 5b and Figure 8b) and u(t) (Figure 6b and Figure 9b) very noisy. Since these signals are directly interfered with noise. The following experiments are aimed at improving the filtering of vector signals ${\dot{x}}_{2}(t)$, u(t). It ensures by additional low-pass filters, with which we will use dynamic generator with parameters (43).

In Experiment 2, we introduce additional block filter on the reference vector signal ${\bar{\chi }}_{1}(t)$ before it enters generator (40):

(44) $$\mu_{1}{\dot{\tau }}_{1}=-\tau_{1}+{\bar{\chi }}_{1}=-\tau_{1}+\chi_{1}+\eta ,\;\;\tau_{1}={\left(\tau_{11},\;\tau_{12},\;\tau_{13}\right)}^{T},\;\;\tau_{1}(0)=\vec{0},$$

where $\mu_{1}=\mathrm{const}>0$ is filter time constant. It is selected to preserve a useful signal $\chi_{1}(t)$ and simultaneously suppress parasitic high-frequency components η(t). Generator (40) with pre-filter (44) has the form:

(45) $$\begin{array}{l} {\dot{x}}_{1}=x_{2},\;\\ {\dot{x}}_{2}=-m_{2}\sigma (k_{2}(x_{2}+m_{1}\sigma (k_{1}(x_{1}-\tau_{1}))))-0.5m_{1}k_{1}\mathrm{diag}\{1-\sigma^{2}(k_{1}(x_{1j}-\tau_{1j}))\}x_{2}. \end{array}$$

**Figure 10:**
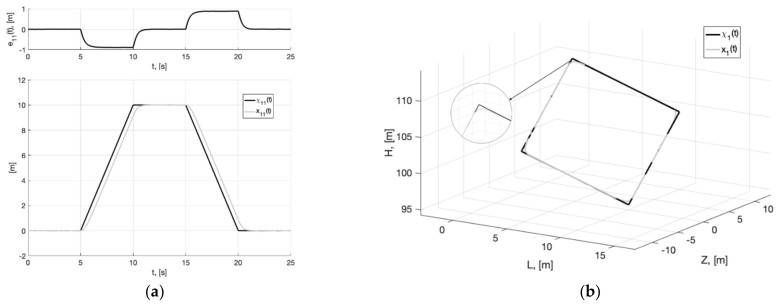
Experiment 2. In (**a**), plots of the reference signal $\chi_{11}(t)$ (38) and its processing of $x_{11}(t)$ (shown below) and tracking error $e_{11}(t)=x_{11}(t)-\chi_{11}(t)$ (shown above). In (**b**), spatial plots of the reference polyline $\chi_{1}(t)$ and point in phase space $x_{1}(t)$ of dynamic generator (45).

**Figure 11:**
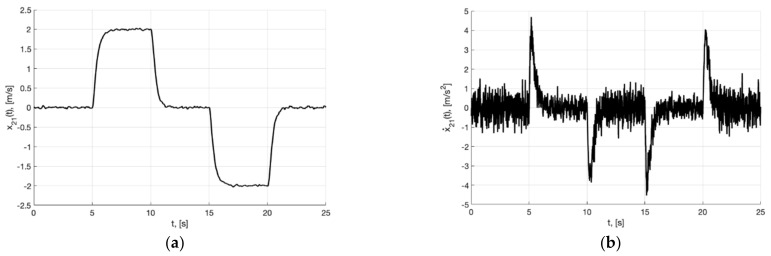
Experiment 2. In (**a**), plot of $x_{21}(t).$ In (**b**), plot of ${\dot{x}}_{21}(t)$ of generator (45).

**Figure 12:**
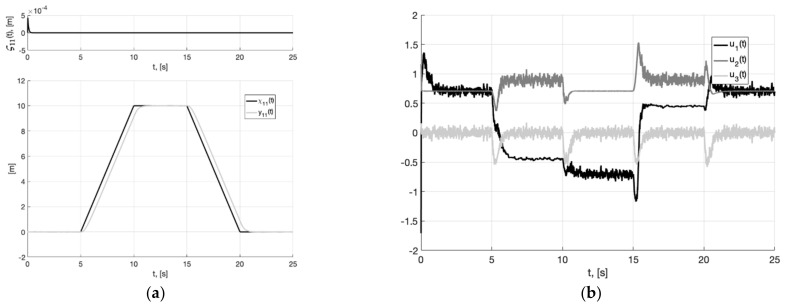
Experiment 2. In (**a**), plots of the reference signal $\chi_{11}(t)$ (38) and output variable $y_{11}(t)$ of closed-loop system (6), (37), (44), (45) (shown below) and tracking error $\varsigma_{11}(t)=y_{11}(t)-x_{11}(t)$ (shown above). In (**b**), plots of the control actions $u_{j}(t),\;j=1,\;2,\;3$ (37).

As a rule, for tuning the filter, the following relations are used [34]:

$$\mu_{1}=1/\omega_{c},\;\;\omega_{c}>\omega ,$$

where $\omega_{c}$ is the desired cutoff frequency (at which the signal power is halved after filtering, and its amplitude is decreased in $\sqrt{2}$ times), ω is the expected frequency of the signal to be filtered. The closer the accepted value $\omega_{c}$ to ω, the more the useful signal is distorted in the neighborhood ω. However, parasitic components are suppressed more strongly at the same time. In contrast, with growth $\omega_{c}$, the useful signal is less distorted. However, the filtering deteriorates. In practice, a compromise is sought based on a priori knowledge of the parasitic component η(t). We accepted $\mu_{1}=10$ for simulation in the experiments below. Figure 10, Figure 11 and Figure 12 show plots of Experiment 2: simulation results of system (6), (37) with pre-filter (44) and generator (45) with parameters (43).

As we can see from Figure 11b and Figure 12b, the parasitic component is decreased significantly in input signals ${\dot{x}}_{21}(t)$ and u(t) compared to Experiment 1. While the output signals and tracking errors (Figure 10a and Figure 12a) are not changed practically. The effects of smoothing corners, boundness of all variables and controls are preserved.

In Experiment 3, the generator (40) still receives a noisy signal $\chi_{1}(t)$. Here, we introduce an additional block filter only on the second derivative ${\dot{x}}_{2}(t)$ before it enters control law (37):

(46) $$\begin{array}{l} \mu_{2}{\dot{\tau }}_{2}=-\tau_{2}+{\dot{x}}_{2}=-\tau_{2}-m_{2}\sigma (k_{2}(x_{2}+m_{1}\sigma (k_{1}(x_{1}-{\bar{\chi }}_{1}))))-\\ -0.5m_{1}k_{1}\mathrm{diag}\{1-\sigma^{2}(k_{1}(x_{1j}-{\bar{\chi }}_{1j}))\}x_{2},\;\;\;\tau_{2}={\left(\tau_{21},\;\tau_{22},\;\tau_{23}\right)}^{T},\;\;\tau_{2}(0)=\vec{0},\; \end{array}$$

where $\mu_{2}=\mathrm{const}>0$ is the filter time constant. In filter (46), in contrast to filter (44), noisy signals are in the arguments of sigmoid functions. Therefore, the area of amplitude changes of the signal to be filtered is obviously bounded. We accepted $\mu_{2}=20$ for simulation in the experiments below.

In a system with a generator (40) and a pre-filter (46), the control law (37) takes the form:

(47) $$u=-B^{T}(C_{1}(y_{1}-x_{1})+C_{2}(y_{2}-x_{2})+ag-\tau_{2})/g.$$

**Figure 13:**
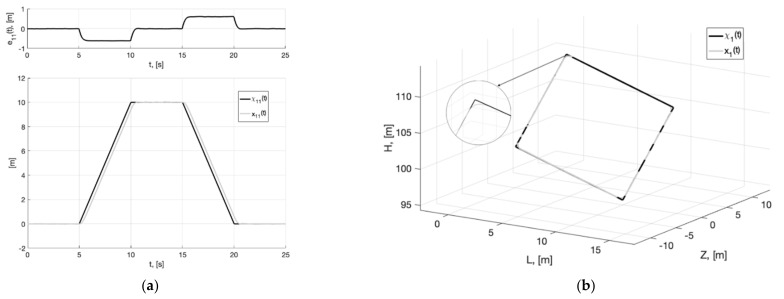
Experiment 3. In (**a**), plots of the reference signal $\chi_{11}(t)$ (38) and its processing of $x_{11}(t)$ (shown below) and tracking error $e_{11}(t)=x_{11}(t)-\chi_{11}(t)$ (shown above). In (**b**), spatial plots of the reference polyline $\chi_{1}(t)$ and point in phase space $x_{1}(t)$ of dynamic generator (40).

**Figure 14:**
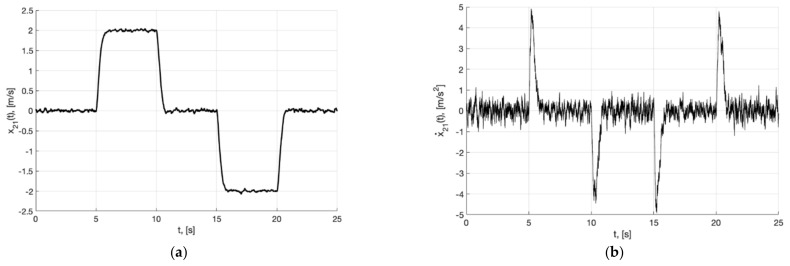
Experiment 3. In (**a**), plot of $x_{21}(t).$ In (**b**), plot of ${\dot{x}}_{21}(t)$ of generator (40).

**Figure 15:**
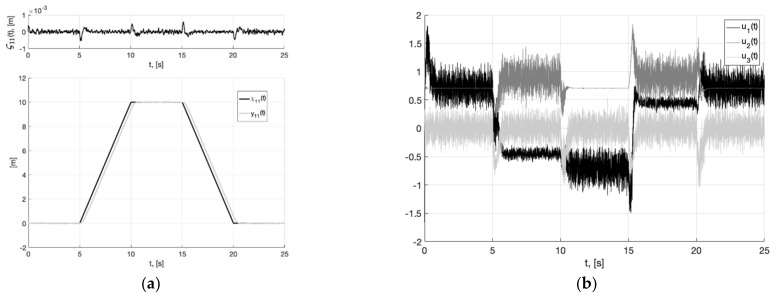
Experiment 3. In (**a**), plots of the reference signal $\chi_{11}(t)$ (38) and output variable $y_{11}(t)$ of closed-loop system (6), (47) (shown below) and tracking error $\varsigma_{11}(t)=y_{11}(t)-x_{11}(t)$ (shown above). In (**b**), plots of the control actions $u_{j}(t),\;j=1,\;2,\;3$ (47).

Figure 13, Figure 14 and Figure 15 present plots of Experiment 3: simulation results of system (6), (47) with pre-filter (46) and generator (40) with parameters (43).

As in Experiment 2, the effects of filtering, smoothing and boundness of signals are preserved. However, the tracking error $e_{11}(t)=x_{11}(t)-\chi_{11}(t)$ turned to be less in Experiment 3 compared to Experiment 2 with the filter on ${\bar{\chi }}_{1}(t)$. Simultaneously, the filter quality of u(t) is worsened.

**Figure 16:**
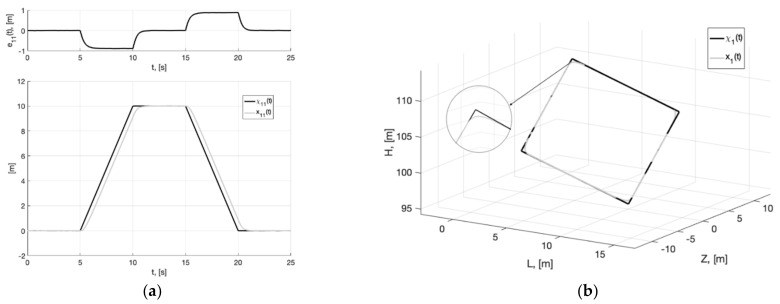
Experiment 4. In (**a**), plots of the reference signal $\chi_{11}(t)$ (38) and its processing of $x_{11}(t)$ (shown below) and tracking error $e_{11}(t)=x_{11}(t)-\chi_{11}(t)$ (shown above). In (**b**), spatial plots of the reference polyline $\chi_{1}(t)$ and point in phase space $x_{1}(t)$ of dynamic generator (45).

**Figure 17:**
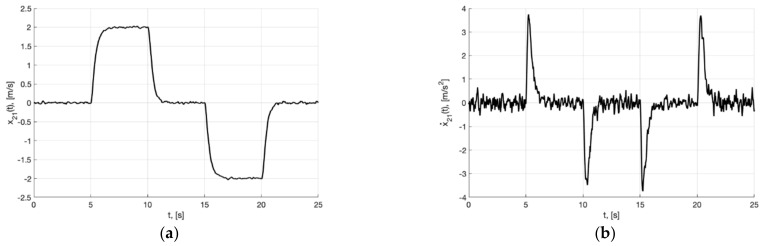
Experiment 4. In (**a**), plot of $x_{21}(t).$ In (**b**), plot of ${\dot{x}}_{21}(t)$ of generator (45).

**Figure 18:**
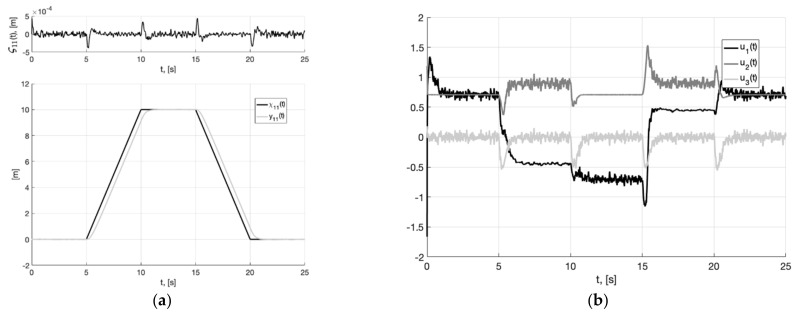
Experiment 4. In (**a**), plots of the reference signal $\chi_{11}(t)$ (38) and output variable $y_{11}(t)$ of closed-loop system (6), (47), (shown below) and tracking error $\varsigma_{11}(t)=y_{11}(t)-x_{11}(t)$ (shown above). In (**b**), plots of the control actions $u_{j}(t),\;j=1,\;2,\;3$ (47).

Finally, Figure 16, Figure 17 and Figure 18 present plots of Experiment 4: simulation results of system (6), (47) with two filters (44) and (46), generator (45) with parameters (43).

Table 2 shows the values of performance indicators of closed-loop systems and signal processing in Experiments 1b and 2–4. We calculated the sample mean (Mean), corrected sample standard deviation (Std) and the maximum value (Max) of the following: absolute value of error $e_{11}(t)=x_{11}(t)-\chi_{11}(t)$, which describes how the generator variable tracks the reference signal; error $\varsigma_{11}(t)=y_{11}(t)-x_{11}(t)$, which describes how the plant output variable tracks the generator variable; absolute value of control $u_{1}(t)$ (for the rest of signals $e_{1}(t),\;\;\varsigma_{1}(t),\;\;u(t)$, the values of indicators are similar). For the calculation, we used the values of the samples from the time intervals indicated in Table 2: for $\varsigma_{11}(t)$ and $u_{1}(t)$ are the first transient response and steady state, for $e_{11}(t)$ are the second one.

We can see from Table 2 that the goal of UAV control was achieved in all experiments: the average value of the error $\varsigma_{11}(t)=y_{11}(t)-x_{11}(t)$ (that show how plant output variable tracks the generator output) is about $10^{-16}$– $10^{-7}$ m (which is due to the presence of noise and numerical integration errors). The use of low-pass filters in Experiments 2–4 reduces the Std of the control action $u_{1}(t)$ by 9.9–49.5 times compared to Experiment 1b, where filters are not used. Note that the values of performance indicators for $e_{11}(t)$ and $u_{1}(t)$ are comparable to those in Experiments 2 and 4. Therefore, additional filtering of the second derivative ${\dot{x}}_{2}(t)$ of the generator is redundant (Experiment 4). It is sufficient to add only one filter on reference signal ${\bar{\chi }}_{1}(t)$ (Experiment 2). The results of Experiments 2 and 3 show that the filtering of the reference signal (Experiment 2) makes it possible to reduce the Std of the control by a factor of 5 compared with filtering the second derivative of the generator (Experiment 3). However, the filter on the reference signal distorts the useful component more. This leads to a larger error $e_{11}(t)$ in Experiment 2 (0.81 m) compared to Experiment 3 (0.58 m). Thus, Experiments 2 and 3 are the most promising for practical implementation. Note that the selection of a specific implementation option depends on the workspace in which the plant operates, the requirements for control smoothness, and the capabilities of computing resources.

We also conducted an additional Experiment 5 to test the performance of the developed approach under time-varying noise parameters (which is close to the real conditions of the UAV operation). The conditions of Experiment 2 were taken as the basis for simulation. We used generator (45) with only one pre-filter (44) for a noisy signal with the same parameters as in Experiment 2. Here, noise η(t) had a normal distribution with a zero mean and time-dependent variance:

$$\mathrm{var}(\eta )=\left\{\begin{array}{c} 0.10,\;0\leq t<5\\ 0.05,\;5\leq t<10\\ 0.02,\;10\leq t<15\\ 0.04,\;15\leq t<20\\ 0.10,\;20\leq t<25 \end{array}\right..$$

We assumed that one knew the maximum admissible value of noise variance, which is 0.1.

**Figure 19:**
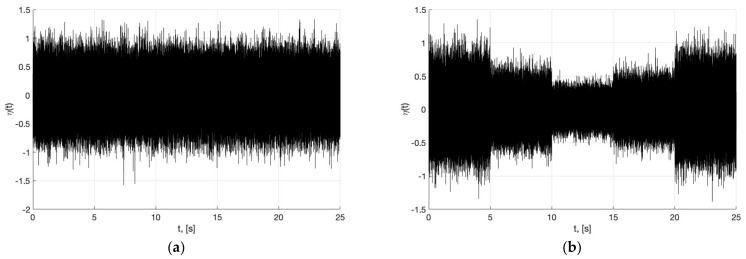
In (**a**), plot of the noise η(t) with constant variance. In (**b**), plot of the noise η(t) when variance is not a constant.

Figure 19 presents a plot of the noise with constant variance used in Experiments 1–4 (on the left side), as well as a plot of the noise with non-constant variance, which we simulated in this Experiment 5 (on the right side).

**Figure 20:**
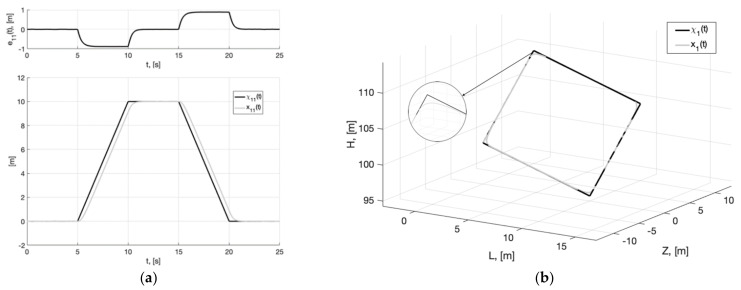
Experiment 5. In (**a**), plots of the reference signal $\chi_{11}(t)$ (38) and its processing of $x_{11}(t)$ (shown below) and tracking error $e_{11}(t)=x_{11}(t)-\chi_{11}(t)$ (shown above). In (**b**), spatial plots of the reference polyline $\chi_{1}(t)$ and point in phase space $x_{1}(t)$ of dynamic generator (45).

**Figure 21:**
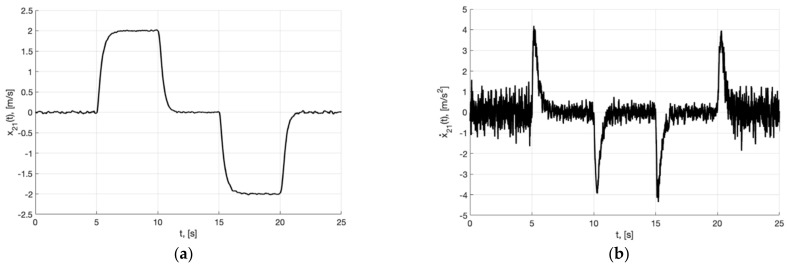
Experiment 5. In (**a**), plot of $x_{21}(t).$ In (**b**), plot of ${\dot{x}}_{21}(t)$ of generator (45).

**Figure 22:**
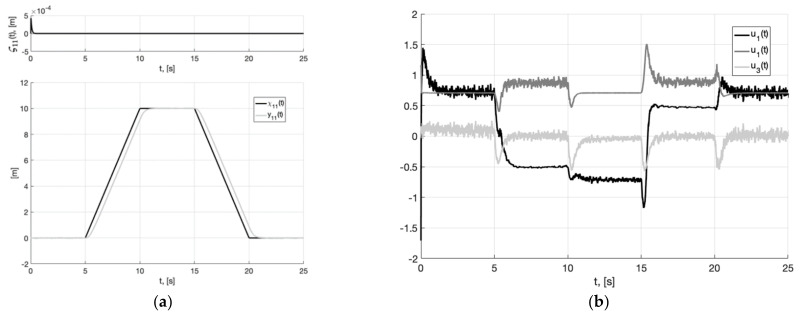
Experiment 5. In (**a**), plots of the reference signal $\chi_{11}(t)$ (38) and output variable $y_{11}(t)$ of closed-loop system (6), (37), (44), (45) (shown below) and tracking error $\varsigma_{11}(t)=y_{11}(t)-x_{11}(t)$ (shown above). In (**b**), plots of the control actions $u_{j}(t),\;j=1,\;2,\;3$ (37).

Figure 20, Figure 21 and Figure 22 show plots of Experiment 5: simulation results of system (6), (37) with pre-filter (44) and generator (45) with parameters (43) in the case of non-constant noise variance.

Thus, we can see from Figure 20, Figure 21 and Figure 22 that the operability of the generator is maintained even in the case of noise parameter variation. To implement the developed method, it is enough to know the limits of their change and the limits of bounded external disturbances, which are considered when tuning the pre-filter and the generator.

## 4. Discussion

The paper was aimed to develop a unified method for differentiating, filtering and smoothing signals for their further use in the UAV control system. This goal was achieved by constructing a dynamic admissible path generator with sigmoid corrective actions. The systems of inequalities are obtained for tuning their parameters in the deterministic case (when there are no noises in the measurements). The ability of a dynamic generator to filter the reference signal and restore its derivatives is not inferior to many existing differentiators. However, in contrast with these, the generator allows us to smooth the signals and consider the constraints on the velocity and acceleration of the UAV. Without loss of generality, the proposed approach can be used to process the reference action signals of control plants, the dynamic model of which is reducible to the canonical form.

The rigorous theoretical calculations in the paper are presented for the deterministic case. The operability of the algorithms in the presence of noise in the measurements has been demonstrated only through numerical simulations. The development of theoretical aspects of the filtering properties of a dynamic generator depending on the noise parameters is the subject for future author’s research.

## Figures and Tables

**Table 1:** The parameters of micro-UAV.

Parameter	Value
Maximum weight, kg	5
Maximum altitude, m	5000
Maximum flight velocity, m/s	26
Flight duration, min	60

**Table 2:** The values of performance indicators of closed-loop systems and signal processing.

Variable	Time Interval	Indicator	Experiment Number			
			1b	2	3	4
$e_{11}(t)$, m	t∈(7; 10)	Mean	0.58	0.81	0.58	0.81
	t∈(7; 10)	Std	0.30	0.31	0.30	0.31
	t∈(5; 10)	Max	1.91	2.11	1.91	2.11
$\varsigma_{11}(t)$, m	t∈(2; 5)	Mean	$1.14\cdot 10^{-14}$	$4.04\cdot 10^{-16}$	$7.54\cdot 10^{-8}$	$3.47\cdot 10^{-7}$
	t∈(2; 5)	Std	$8.55\cdot 10^{-16}$	$4.68\cdot 10^{-17}$	$5.30\cdot 10^{-5}$	$3.16\cdot 10^{-5}$
	t∈(0; 5)	Max	$4.30\cdot 10^{-4}$	$4.30\cdot 10^{-4}$	$3.97\cdot 10^{-4}$	$4.34\cdot 10^{-4}$
$u_{1}(t)$	t∈(2; 5)	Mean	0.71	0.71	0.71	0.71
	t∈(2; 5)	Std	0.99	0.02	0.10	0.02
	t∈(0; 5)	Max	3.12	1.36	1.71	1.35
